# A new wine *Torulaspora delbrueckii* killer strain with broad antifungal activity and its toxin-encoding double-stranded RNA virus

**DOI:** 10.3389/fmicb.2015.00983

**Published:** 2015-09-15

**Authors:** Manuel Ramírez, Rocío Velázquez, Matilde Maqueda, Antonio López-Piñeiro, Juan C. Ribas

**Affiliations:** ^1^Departamento de Ciencias Biomédicas (Área de Microbiología, Antiguo Rectorado), Facultad de Ciencias, Universidad de ExtremaduraBadajoz, Spain; ^2^Departamento de Biología Vegetal, Ecología y Ciencias de la Tierra, Facultad de Ciencias, Universidad de ExtremaduraBadajoz, Spain; ^3^Instituto de Biología Funcional y Genómica, CSIC/Universidad de SalamancaSalamanca, Spain

**Keywords:** wine, yeast, *Torulaspora*, killer, virus, dsRNA

## Abstract

Wine *Torulaspora delbrueckii* strains producing a new killer toxin (Kbarr-1) were isolated and selected for wine making. They killed all the previously known *Saccharomyces cerevisiae* killer strains, in addition to other non-*Saccharomyces* yeasts. The Kbarr-1 phenotype is encoded by a medium-size 1.7 kb dsRNA, TdV-Mbarr-1, which seems to depend on a large-size 4.6 kb dsRNA virus (TdV-LAbarr) for stable maintenance and replication. The TdV-Mbarr-1 dsRNA was sequenced by new generation sequencing techniques. Its genome structure is similar to those of *S. cerevisiae* killer M dsRNAs, with a 5′-end coding region followed by an internal A-rich sequence and a 3′-end non-coding region. Mbarr-1 RNA positive strand carries *cis* acting signals at its 5′ and 3′ termini for transcription and replication respectively, similar to those RNAs of yeast killer viruses. The ORF at the 5′ region codes for a putative preprotoxin with an N-terminal secretion signal, potential Kex2p/Kexlp processing sites, and N-glycosylation sites. No relevant sequence identity was found either between the full sequence of Mbarr-1 dsRNA and other yeast M dsRNAs, or between their respective toxin-encoded proteins. However, a relevant identity of TdV-Mbarr-1 RNA regions to the putative replication and packaging signals of most of the M-virus RNAs suggests that they are all evolutionarily related.

## Introduction

The *Saccharomyces cerevisiae* killer strains have been grouped so far into four types (K1, K2, K28, and Klus) based on their killing profiles and lack of cross-immunity. Members of each group can kill non-killer yeasts as well as killer yeasts belonging to the other killer yeast types. These killer yeasts secrete protein toxins that are also lethal to other yeast species (Rodríguez-Cousiño et al., 2011). Each killer yeast is immune to its own toxin or to toxins produced by strains of the same killer type (Schmitt and Breinig, 2006). *S. cerevisiae* killer toxins (K1, K2, K28, and Klus) are encoded by the positive strand of medium-size (1.6–2.4 kb) dsRNA of yeast viruses (M1, M2, M28, and Mlus, respectively). The RNA 5′-end region contains an ORF that codes for the toxin precursor or preprotoxin (pptox), which also provides immunity to its own killer toxin. The four toxin-coding M dsRNAs show no sequence identity with each other (Schmitt and Tipper, 1995; Rodríguez-Cousiño et al., 2011). These M viruses depend on a second large-size (4.6 kb) dsRNA helper virus, LA, for maintenance and replication. LA provides the capsids and polymerase in which both LA and M dsRNAs are separately encapsidated and replicated, and the M dsRNAs contain some stem-loop structures that mimic those LA dsRNA signals required for genome packaging or replication [reviewed by Schmitt and Breinig (2006)]. The LA genome code for two proteins, the major coat protein Gag and a minor Gag-Pol fusion protein translated by a -1 ribosomal frameshifting mechanism and that contains all the activities required for virus propagation (Icho and Wickner, 1989; Dinman and Wickner, 1992; Fujimura et al., 1992; Park et al., 1996).

The *cis* signals required for RNA packaging and replication are located in the 3′-terminal regions of the positive strands of both LA and M RNAs (Wickner et al., 1995; Rodríguez-Cousiño et al., 2011). The signal for transcription initiation has been proposed to be present in the 5′-end first 25 nucleotides of LA RNA, probably in the very 5′-terminal sequence itself (5′-GAAAAA). This 5′-terminal recognition element is also present in the 5′ end of M1, M2, M28, and Mlus RNAs (Fujimura et al., 1990; Rodríguez-Cousiño et al., 2011). The M1, M2, or M28 ORF is translated into a pptox that subsequently enters the secretory pathway for further processing and secretion as a mature toxin. The unprocessed pptox consists of an N-terminal signal sequence necessary for its import into the endoplasmic reticulum lumen, followed by the α- and β-subunits of the mature toxin separated from each other, in the case of K1 and K28, by a potentially N-glycosylated γ-sequence. The signal peptide is removed in the endoplasmic reticulum, and N-glycosylation and disulphide bond formation occurs. Then, in a late Golgi compartment, protease processing takes place involving Kex2 and Kex1 proteases. Finally, the toxin is secreted as an active α/β heterodimer, with the two subunits being covalently linked by one or more disulphide bonds (Wickner et al., 1995; Schmitt and Breinig, 2006). All this processing is also believed to occur in Klus pptox according to its predicted amino acid sequence (Rodríguez-Cousiño et al., 2011). There are some other non-*Saccharomyces* killer yeasts containing a similar set of dsRNA helper and satellite viruses responsible for their killer phenotype, such as *Hanseniaspora uvarum*, *Zygosaccharomyces bailii*, or *Ustilago maydis* (Magliani et al., 1997).

*Torulaspora delbrueckii* was one of the first non-*Saccharomyces* yeasts to be released onto the market, and is probably the most used for winemaking. Controlled inoculation with this yeast is broadly recommended for improving the complexity and enhancing some specific characteristics of wines (Jolly et al., 2006; Azzolini et al., 2015). However, its impact on wine quality is still far from satisfactory, mostly because of the difficulty in controlling the desired participating proportion of *T. delbrueckii* with respect to the other wine yeast species involved in the same must fermentation process, mainly *S. cerevisiae*-like yeasts. As with most non-*Saccharomyces* yeasts, *T. delbrueckii* has less fermentation vigor and slower growth rate than *S. cerevisiae* under usual wine fermentation conditions, being quickly overcome by wild or inoculated *S. cerevisiae* strains (González-Royo et al., 2014). Additionally, cell-to-cell contact has also been suggested to explain this decline of *T. delbrueckii* when it is in the presence of *S. cerevisiae* (Nissen et al., 2003; Renault et al., 2013). Thus, knowledge of the *Saccharomyces* and *Torulaspora* wine yeast interactions during wine fermentation needs to be improved to better control each yeast’s participation rate (Ciani et al., 2010). The availability of good-fermenting *T. delbrueckii* killer strains, able to kill the omnipresent wild *Saccharomyces* yeasts, would be an interesting tool to achieve the inoculated yeast’s dominance of the must fermentation process, so that the result would be the desired improvement in quality of the wine. The isolation of *T. delbrueckii* killer strains has been described (Sangorrín et al., 2007a,b), but no detailed phenotype or genotype analysis of these killer strains has as yet been reported.

The objective of the present work was the phenotypic analysis and genotypic characterization of new *T. delbrueckii* killer strains (Kbarr-1) isolated from the *Barraecas* valley in Spain. We addressed the following questions: (i) antifungal spectrum of the Kbarr-1 toxin, (ii) isolation, sequencing, and characterization of Mbarr-1 dsRNA satellite viruses, and (iii) analysis of Mbarr-1 genome organization and its preprotoxin ORF as compared with the dsRNA of other M viruses. The possible evolutionary relationship between these groups of viral M dsRNAs is discussed.

## Materials and Methods

### Yeast Strains and Media

The new *T. delbrueckii* Kbarr wine yeasts are prototrophic strains isolated from spontaneous fermentations of grapes from vineyards of the Albarregas (*Barraecas* in Latin) river valley in Spain. The industrial use of these Kbarr yeasts is under patent application. The yeast strains used in this work are summarized in **Table 1**.

**Table 1:** Yeast strains used in this study.

Strain	Genotype/relevant phenotype	Origin
*Sc* EX88	*MATa/*α *HO/HO cyh^*S*^/cyh^*S*^* [K2^+^]	M. Ramírez^a^ (from wine)
*Sc* EX85	*MAT a/α HO/HO* L-A M-2[K2^0^]	M. Ramírez^a^ (from wine)
*Sc* EX85R	*MAT a/α HO/HO CYH^*R*^/cyh^*S*^* [cyh^R^ K2^0^]	M. Ramírez^a^
*Sc* E7AR1	*MAT a/α HO/HO CYH^*R*^/cyh^*S*^* [K2^+^]	M. Ramírez^a^
*Sc* Rod23-1B	*MAT a/α HO/HO PDR5/pdr5*[Rod^PC^ K2^+^]	M. Ramírez^a^
*Sc* EX229	*MAT a/α HO/HO* L-A M-lus [Klus^+^]	M. Ramírez^a^ (from wine)
*Sc* EX229-R1	*MAT a/α HO/HO CYH^*R*^/cyh^*S*^* [cyh^R^ Klus^0^]	M. Ramírez^a^
*Sc* EX33	*MATa/*α *HO/HO* [L-A^0^ K1^0^ K2^0^ K28^0^ K*lus*^0^]	M. Ramírez^a^ (from wine)
*Sc* EX73	*MATa/*α *HO/HO* L-A M2 [K2^+^]	M. Ramírez^a^ (from wine)
*Sc* F166	*MAT*α *leu1 kar1* L-A-HNB M1 [K1^+^]	J.C. Ribas^b^ (from R. B. Wickner)
*Sc* F182	*MAT*α *his2 ade1 leu2-2 ura3-52 ski2-2* L-A M28 [K28^+^]	J. C. Ribas^b^ (from M. Schmitt)
*Sc* EX198	*MATa/*α *HO/HO* L-A M*lus* [K*lus*^+^]	M. Ramírez^a^ (from wine)
*Td* TD291	*wt* L-A [K2^0^]	Lallemand Inc.^c^
*Td* EX1180	*wt* L-A Mbarr-1 [Kbarr-1^+^]	This study (from wine)
*Td* EX1180-11C4	*cyh^*R*^* L-Abarr Mbarr-1 [cyh^R^ Kbarr-1^+^]	This study (from EX1180)
*Td* EX1180-2K^-^	*cyh^*R*^* L-Abarr Mbarr-0 [cyh^R^ Kbarr^0^]	This study (from EX1180)
*Td* EX1257	*wt* L-Abarr Mbarr-2 [Kbarr-2^+^]	This study (from wine)
*Td* EX1257-CYH5	*cyh^*R*^* L-Abarr Mbarr [cyh^R^ Kbarr-2^+^]	This study (from EX1257)
*Candida albicans* 10231	Pathogen. 87% of membrane hydrophobicity at 37°C, and 4% at 22°C	C. López^d^
*C. kefir*	Pathogen	C. López^d^
*C. glabrata*	Pathogen	C. López^d^
*C. dubliniensis*	Pathogen	C. López^d^
*C. krusei*	Pathogen	C. López^d^
*C. parasilopsis*	Pathogen	C. López^d^
*C. tropicalis*	Pathogen	C. López^d^
*C. albicans* wt 5314C	Pathogen	J. Correa^e^
*C. albicans* CAF	*(wt URA3+/-)*/Pathogen	J. Correa^e^
*Yarrowia lipolytica* wt *a*		L.M. Hernández^f^
*Y. lipolytica mnn9 a*	Mutation *mnn9* truncates carbohydrate outer chain of the cell wall mannoproteins of *S. cerevisiae*	L.M. Hernández^f^
*Y. lipolytica* SA1-5 wt		A. Domínguez^g^
*Kluyvermyces lactis*	Killer phenotype encoded in a dsDNA plasmid (pGKL1)	A. Domínguez^g^
*Hansenula mrakii* wt	Killer, toxin HM1 encoded in a chromosomal gene	J.C. Ribas^b^ (from T. Watanabe)
*Schizosaccharomyces pombe* wt 972h^-^		J.C. Ribas^b^
*Hanseniaspora* sp.	Killer against *S. cerevisiae*	M. Ramírez^a^ (from wine)
*Brettanomyces* sp.		M. Ramírez^a^ (from wine)

*^a^M. Ramírez, Departamento de Ciencias Biomédicas, Universidad de Extremadura, Badajoz, Spain.*

*^b^J. C. Ribas, Instituto de Biología Funcional y Genómica, CSIC/Universidad de Salamanca, Salamanca, Spain.*

*^c^Lallemand Inc., 19 Rue des Briquetiers, BP 59, 31702 Blagnac Cedex, France.*

*^d^C. López, Departamento de Ciencias Biomédicas, Universidad de Extremadura, Badajoz, Spain.*

*^e^J. Correa, Departamento de Ciencias Biomédicas, Universidad de Extremadura, Badajoz, Spain.*

*^f^L. M. Hernández, Departamento de Ciencias Biomédicas, Universidad de Extremadura, Badajoz, Spain.*

*^g^A. Domínguez, Departamento de Microbiología y Genética, Universidad de Salamanca, Salamanca, Spain.*

*Sc, Saccharomyces cerevisiae; Td, Torulaspora delbrueckii.*

Standard culture media were used for yeast growth (Guthrie and Fink, 1991). YEPD contained 1% yeast extract, 2% peptone, and 2% glucose. YEPD+cyh is YEPD supplemented with cycloheximide (cyh) to a final concentration of 2 μg/ml. Synthetic minimal medium (SD) contained 0.67% Yeast Nitrogen Base (without amino acids; with ammonium sulfate, Difco), and 2% glucose. The corresponding solid media also contained 2% agar. Standard procedures were used for sporulation of yeast cultures (Kaiser et al., 1994). Diploid cells grown on YEPD plates for 2 days at 30°C were transferred to sporulation plates (1% potassium acetate, 0.1% yeast extract, 0.05% glucose, 2% agar) and incubated for 7–30 days at 25°C until cells sporulated.

### Determination of Yeast Killer Activity

Killer activity was tested on low-pH (pH 4.0 or 4.7) methylene blue plates (4 or 4.7 MB; Kaiser et al., 1994) seeded with 100 μl of a 48-h grown culture of the sensitive strain (Ramírez et al., 2004). Depending on the experiments, the strains being tested for killer activity were either loaded as 4 μl drops of stationary phase cultures, patched from solid cultures, or replica plated onto the seeded MB plates. Then the plates were incubated for 4–8 days at 12 or 20°C.

### Total Nucleic Acid Preparation and Nuclease Digestion

The procedure for routine dsRNA and mitochondrial DNA (mtDNA) minipreps was described previously (Maqueda et al., 2010). Basically, the cells were suspended in 10 mM Tris-HCI (pH 7.5) buffer containing 0.1 M NaCl, 10 mM EDTA and 0.2% SDS, thereafter, an equal volume of phenol (pH 8.0) was added. This mixture was incubated at room temperature for 30 min with shaking. After centrifugation, the nucleic acids recovered in the aqueous phase were precipitated with isopropanol, washed with 70% ethanol, dried, and dissolved in TE buffer pH 8.0. Digestion of DNA was done with DNAse I (RNAse-free, Fermentas Life Sciences) according to the manufacturer’s specifications. Digestion of RNA was performed with RNAse A (Sigma-Aldrich) following the manufacturer’s indications. For selective degradation of single-stranded RNA, samples were incubated with RNAse A (10 μg/ml) in the presence of 0.5 M NaCl for 30 min at 37°C. Samples were then processed through phenol/chloroform/isoamyl alcohol extraction to inactivate the enzyme before analysis through agarose gel electrophoresis (Rodríguez-Cousiño et al., 2011).

### Nucleic Acid Analysis for Yeast Strain Typing and Identification of *Torulaspora* Species

The procedure for virus dsRNA analysis has been described previously (Maqueda et al., 2010). The samples (4 μl) were directly separated in 1 × TAE-1% agarose gel electrophoresis for virus dsRNA analysis. Nucleic acids were visualized on a UV transilluminator after ethidium bromide staining of the gels and photographed with a Gel Doc 2000 (Bio-Rad). The data analysis was performed using Diversity Database software (Bio-Rad). The nucleic acid bands were typed by Rf, and band assignment was determined by Rf values plus or minus 2% error. The identification of *Torulaspora* species by restriction fragment length polymorphism (RFLP) analysis of the internal transcribed spacers (ITS) of ribosomal DNA was done as described previously (Esteve-Zarzoso et al., 1999).

### PCR Amplification, Sequencing of 18S Ribosomal DNA (rDNA), and Yeast Identification

The PCR was performed directly from the nucleic acid minipreps with the kit pReTaq Ready-To-Go PCR Beads (Amersham Biosciences), with the 18S rDNA specific primers EukA (AACCTGGTTGATCCTGCCAGT) and EukB (TGATCCTTCTGCAGGTTCACCTAC; Medlin et al., 1988; Díez et al., 2001). The thermocycler protocol was an initial denaturation step of 95°C for 2 min, followed by 35 cycles of denaturing at 95°C for 15 s, annealing at 55°C for 15 s, and extension at 72°C for 2 min; and a final extension at 72°C for 10 min. The amplification products were purified with the “Jetquick PCR purification Spin Kit” (Genomed) following the manufacturer’s recommendations. The purified rDNA PCR fragment from each isolated microorganism was sent to a sequencing service (Secugen, Madrid, Spain). The 18S rDNA gene sequences were edited with the software Chromas v. 1.45^1^, and analyzed against those in GenBank using Blast (Altschul et al., 1990). Sequences of >99% similarity to previously published data available at NCBI^2^ were binned into the same species.

### Viral dsRNA Purification

Total nucleic acid preparation from *T. delbrueckii* EX1180 strain (killer Kbarr-1) was obtained by the procedures mentioned above (Maqueda et al., 2010). After 1% agarose gel electrophoresis, the slower-moving dsRNA band (4.6 kb) and the faster-moving dsRNA band (1.7 kb) were cut off from the gel and purified with RNaid Kit (Q-Biogene) following the manufacturer’s indications. This procedure was repeated until more than 20 μg of each purified dsRNA was obtained.

### Preparation and Sequencing of cDNA Libraries from Purified Viral dsRNA

The purified dsRNA samples were sent to the Unidad de Genómica Cantoblanco (Fundación Parque Científico de Madrid, Spain) for cDNA library preparation and next generation sequencing (NGS). Libraries from TdV-Mbarr-1 (1.7 kb dsRNA purified band) were prepared with the “TruSeq RNA Sample Preparation kit” (Illumina) following the company’s instructions, and using 200 ng of purified dsRNA as input (quantified with Picogreen). Briefly, this protocol started at the fragmentation step, skipping the RNA purification step as the viral dsRNA had been previously purified as mentioned above. Thereafter, 15% DMSO was added to the Illumina fragment-prime solution before incubation at 94°C for 8 min to facilitate the dsRNA denaturation. The first strand of cDNA was synthesized using random primers, dTVN and dABN oligonucleotides (Isogen Life Science), and SuperScriptIII retrotranscriptase. The dTVN and dABN oligonucleotides were added to improve the retrotranscription of the expected central poly(A) region of the M virus. Thereafter, the second cDNA strand synthesis, end repair, 3′-ends adenylation, and ligation of the TruSeq adaptors were done (Illumina). These adaptor oligonucleotides included signals for further amplification and sequencing, and also included short sequences referred to as indices which allowed multiplexing in the sequencing run. An enrichment procedure based on PCR was then performed to amplify the library, ensuring that all molecules in the library included the desired adaptors at both ends. The number of PCR cycles was adjusted to 12 and the final amplified libraries were checked on a BioAnalyzer 2100 (Agilent Technology). The libraries were denatured prior to seeding on a flow cell, where clusters were formed, and sequenced using 2 × 80–2 × 150 sequencing runs on a MiSeq instrument.

### dsRNA Sequence Assembling

The obtained cDNA sequences were analyzed and assembled by Biotechvana (Technological Park of Valencia, Spain). As no high-identity reference sequence was available for TdV-Mbarr-1 sequence assembly, its full-length sequence was reconstructed using the following strategy. First, SOAP deNOVO2 (Luo et al., 2012) was used to obtain a *de novo* assembly based on two Illumina libraries, trying multiple assembly attempts with scaffolding and insert size of 200 and varying the Kmer value (with 47 being the most effective Kmer in reported sequences with a minimal number of undetermined positions filled with “Ns”). This K47 assembly comprised several contigs and scaffolds, none of them showing the expected typical features of viral M RNAs such as a 5′ GAAAA-like motif or a large poly(A) trait in the middle of the sequence. To filter the sample, contigs with size shorter than 300 nucleotides were removed from the contig file, while the remaining contigs were used as input to the NR database of the NCBI via the BLASTX search protocol (Altschul et al., 1997) implemented in the software GPRO 1.1 (Futami et al., 2011). The BLASTX results did not report similarity to any viral M-like RNA sequence described in the scientific literature; conversely, highly significant similarity was found between several contigs/scaffolds and some known viral RNA sequences (LA, LBC, and others) or host transcripts. These supposed contaminating sequences were also filtered from the assembly. Assuming that our Mbarr-1 sequence may have resulted fragmented during the assembly procedure because of the low complexity in the middle of the sequence containing a putative central poly(A), a manual procedure for sequence assembly was used as follows. Bowtie2 (Langmead et al., 2009) and two *ad hoc* selected scripts were used to filter all raw reads corresponding to the contaminant sequences by mapping the two Illumina libraries against the contaminant sequences. Then we manually searched the new cleansed Illumina libraries for new raw reads containing a prominent central poly(A) trait, or its complementary poly(T), to manually construct contigs based on at least 10 reads containing the poly(A). Interestingly, only one contig passed this filter. This contig was enlarged by aligning other reads that made a contig with its 5′ and 3′ ends. In doing so, a manually constructed contig of 300 nucleotides with a central 78-mer poly(A), and well defined and informative 5′ and 3′ ends, was obtained. We then searched the K47 assembly for sequences matching these two 5′ and 3′ ends of the poly(A) contig with at least 100 nucleotides. Two contigs of the K47 assembly were identified under this strategy, and used to resolve a contig consisting in an almost full-length sequence of TdV-Mbarr-1 RNA containing approximately 1500 nucleotides. This sequence was also enlarged as much as possible by selecting again other raw reads (from the cleansed Illumina libraries) making a contig with both the 5′ and 3′ ends of the reconstructed sequence. The final sequence resulted in a 1705 nucleotide contig showing the expected 5′ GAAAAA and the typical domain architecture of viral M RNAs. Finally, Bowtie2 (Langmead and Salzberg, 2012) was used to map the cleansed Illumina libraries over the reconstructed final contig in order to obtain a consensus sequence of TdV-Mbarr-1 RNA genome. Visual navigation of the resulting BAM file with the IGV browser (Thorvaldsdottir et al., 2013) showed full coverage of the whole RNA sequence thus validating the methodological approach taken in reconstructing the full-length Mbarr-1 RNA genome.

### Miscellaneous

DNA manipulations (enzyme digestions, PCR, and electrophoresis) were done following standard methods according to (Sambrook et al., 1989). Most of the enzymes were from Promega or Sigma. Synthetic oligonucleotides were from Biomers.

### Nucleotide Sequence Accession Number

The TdV-Mbarr-1 cDNA nucleotide sequence and the encoded Mbarr-1 protein appear in NCBI/GenBank under GenBank accession number KT429819.

## Results

### Phenotypic Characterization of Kbarr Killer Yeasts

**FIGURE 1:**
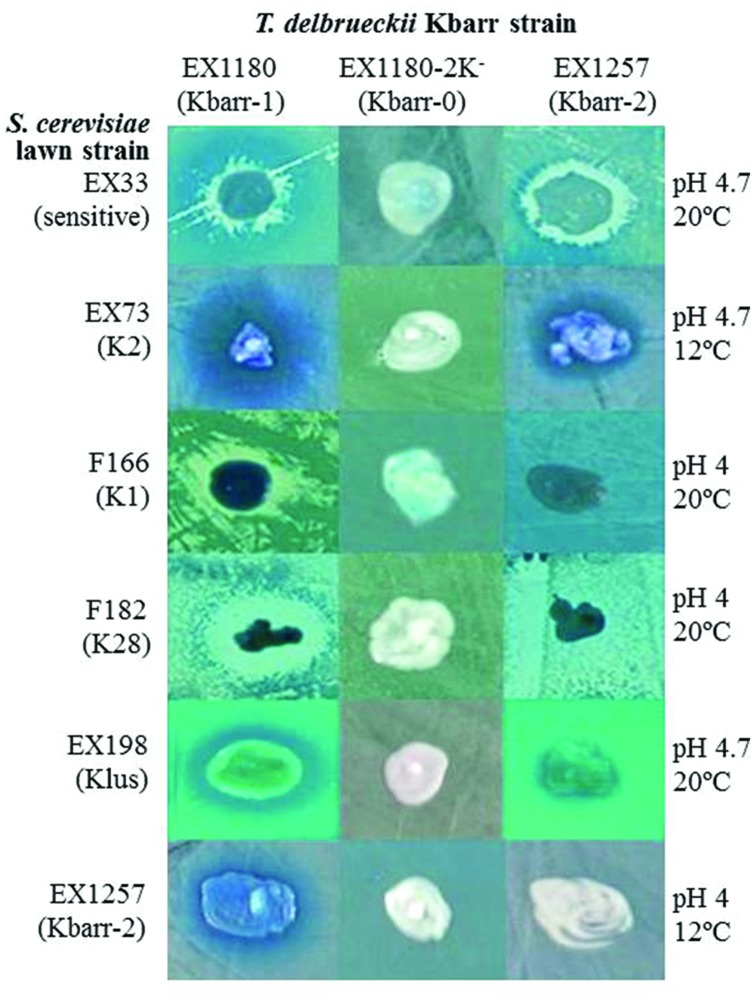
**Killer phenotype assay of Kbarr-1 strain EX1180, Kbarr-2 strain EX1257, and Kbarr-0 strain EX1180-2K^-^ (from EX1180 after cyh treatment) of *Torulaspora delbrueckii* wine yeasts against *Saccharomyces cerevisiae* and *T. delbrueckii* strains.** The assay was done on methylene blue agar plates seeded with standard *S. cerevisiae* killer-sensitive (EX33) or killer K2 (EX73), K1 (F166), K28 (F182), and K*lus* (EX198) strains and *T. delbrueckii* Kbarr-2 (EX1257) strain. The assay conditions (pH and temperature) are given on the right.

**FIGURE 2:**
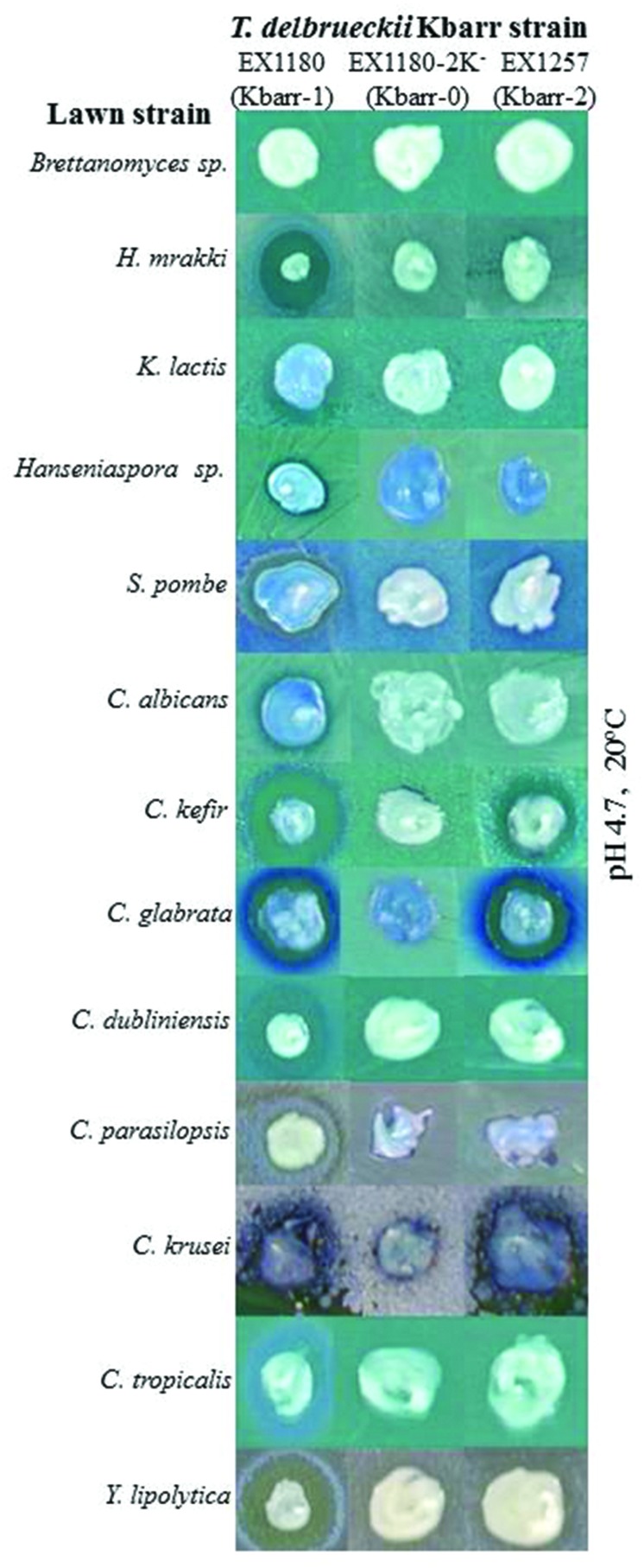
**Killer phenotype assay of Kbarr-1 strain EX1180, Kbarr-2 strain EX1257, and Kbarr-0 strain EX1180-2K^-^ of *T. delbrueckii* wine yeasts against non-*Saccharomyces* yeasts (indicated on the left).** The assay was done at pH 4.7 and 20°C.

We analyzed the killer phenotype of 1264 *Saccharomyces*-like colonies isolated from 112 spontaneous fermentations of grapes collected from different vineyards of the “Ribera del Guadiana” region (Spain) during several vintages. The killer strains were compared to the previously known *S. cerevisiae* K1, K2, K28 and Klus killer strains, and the EX33 non-killer strain (**Table 1**). New killer yeasts were found only in two fermentations of grapes from the Albarregas (*Barraecas* in Latin) river valley and named Kbarr killer strains. These Kbarr yeasts remained in more than 50% of the total viable yeast cells at the tumultuous or end fermentation stages. Two types of Kbarr phenotypes were found, Kbarr-1 and Kbarr-2. The Kbarr-1 strains killed the Kbarr-2 (**Figure 1**) strains, while the Kbarr-2 strains did not kill the Kbarr-1 strains (not shown). Two Kbarr-1 yeast isolates (EX1178 and EX1180) were selected for wine making according to the criteria described previously (Regodón et al., 1997), and therefore chosen for deeper killer phenotype and genotype analyses. Initially, no Kbarr-2 yeast isolate was chosen for such analysis because they showed lower killer activity than Kbarr-1 strains (**Figures 1** and **2**). All killer Kbarr isolates were prototrophic yeasts identified as *T. delbrueckii* according to their physiological characteristics (Kurtzman, 2011), 18S ribosomal RNA sequence (Maqueda et al., 2012), and ITS-RFLPs (Esteve-Zarzoso et al., 1999) analyses.

The Kbarr-1 strains killed the four known K1, K2, K28, and Klus killer strains of *S. cerevisiae* and the aforementioned *T. delbrueckii* Kbarr-2 strain, but they did not kill other Kbarr-1 yeasts (**Figure 1**). They were also lethal to yeast species other than *S. cerevisiae*, such as *Hanseniaspora* sp., *Kluyveromyces lactis*, *Schizosaccharomyces pombe*, *Candida albicans*, *C. tropicalis*, *C. dubliniensis*, *C. kefir*, *C. glabrata*, *C. parasilopsis*, *C. krusei*, *Yarrowia lipolytica*, and *Hansenula mrakii*, although unfortunately they did not kill the wine spoilage yeast *Brettanomyces* as it might have been desired (**Figure 2**). The Kbarr-1 strains were only weakly sensitive to killer toxins produced by *S. cerevisiae* Klus and *H. mrakii*, and resistant to the rest of the tested killer yeasts (not shown). The killing-activity spectrum of the Kbarr-1 yeasts was even broader than that of the recently discovered *S. cerevisiae* Klus yeasts (Rodríguez-Cousiño et al., 2011). Kbarr-1 yeasts showed stronger killer activity than K2 or Klus yeasts, and similar activity to those of *S. cerevisiae* K1, or K28 strains. The Kbarr-1’s strongest activity was found at pH 4.7 and 12°C against *S. cerevisiae* K2, pH 4 and 20°C against *S. cerevisiae* K28, and pH 4.7 and 20°C against *H. mrakii*, *C. kefir*, *C. glabrata*, *C. dubliniensis*, and *Y. lipolytica* strains (**Figures 1** and **2**).

### Genotypic Characterization of Kbarr-1 Killer Yeasts

**FIGURE 3:**
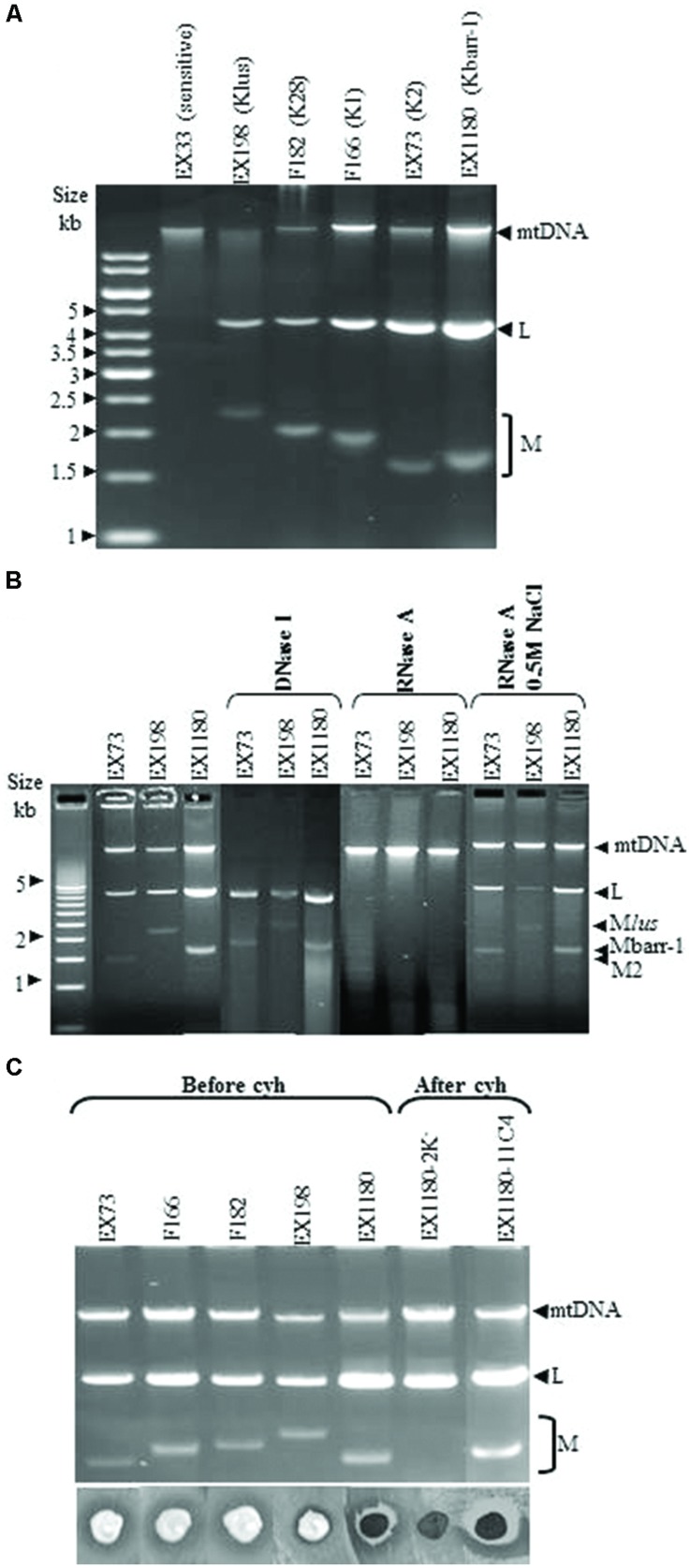
**Genetic determinants of Kbarr-1 phenotype. (A)** Presence of L and M molecules in Kbarr-1 strains. Nucleic acids were obtained from sensitive (EX33), Klus (EX198), K28 (F182), K1 (F166), K2 (EX73), and Kbarr-1 (EX1180) strains, and separated by agarose gel electrophoresis. The ethidium bromide staining of the gel is shown. **(B)** Nuclease treatments. Total nucleic acids from strains K2 (EX73), Klus (EX198), and Kbarr-1 (EX1180), after DNAse I digestion, or after RNAse A treatment under low or high salt conditions, were separated by agarose gel electrophoresis. **(C)** Cycloheximide curing of killer Mbarr-1 virus. Agarose gel electrophoresis of total nucleic acids from killer K2 (EX73), K1 (F166), K28 (F182), Klus (EX198), and Kbarr-1 (EX1180) strains before virus curing with cycloheximide and after treatment with cycloheximide of two EX1180 clones losing or maintaining the M dsRNA (top panel). The killer phenotype assay is shown (bottom panel). EX1180-2K^-^ is a cycloheximide-cured clone from EX1180, and EX1180-11C4 is a cycloheximide-non-cured clone from EX1180. The assay was done on methylene blue agar plates (pH 4, 20°C) seeded with the *S. cerevisiae* K28 strain (F182) for the *T. delbrueckii* killer assay, or the *S. cerevisiae* K-0 sensitive strain EX33 for the rest of the *S. cerevisiae* killer assays.

All Kbarr-1 killer strains carried two nucleic acid bands that showed an agarose-gel-electrophoresis mobility similar to those of viral dsRNAs from other killer yeasts: (1) a slower-moving band, similar in size to the dsRNA genome of ScV-LA virus (4.6 kb, now named the TdV-LAbarr band); and (2) a faster-moving band, similar to the dsRNA genome of ScV-M viruses (1.7 kb, now named the TdV-Mbarr-1 band; **Figure 3A**). The dsRNA nature of the two nucleic acid bands was confirmed by DNAse I and RNAse A treatments. The mtDNA disappeared after DNAse I treatment, while LA, M2, and Mlus dsRNAs used as controls, and the bands of similar sizes present in the Kbarr-1 strains, remained unaffected (**Figure 3B**). Additionally, LA, M2, Mlus dsRNA bands and the Kbarr-1 strain bands disappeared after RNAse A treatment, while mtDNA remained unaffected. Moreover, the RNA molecules were fairly resistant to RNAse A digestion in the presence of 0.5 M NaCl, as expected for dsRNA but not for ssRNA (Berrye and Bevane, 1972; Vodkin and Fink, 1973; Cansado et al., 1999; **Figure 3B**). Mbarr-1 dsRNA was lost during the growth of Kbarr-1 strains in the presence of cycloheximide, and a concomitant loss of its killer activity was observed (**Figure 3C**). This result suggests that the Kbarr-1 killer phenotype is encoded by the Mbarr-1 dsRNAs, as has been previously described for the killer toxins encoded by M1, M2, M28, and Mlus dsRNAs in *S. cerevisiae* (Berrye and Bevane, 1972; Vodkin and Fink, 1973; Wickner, 1991; Rodríguez-Cousiño et al., 2011).

### Analysis of TdV-Mbarr-1 dsRNA Sequence and Kbarr-1 Preprotoxin ORF

**FIGURE 4:**
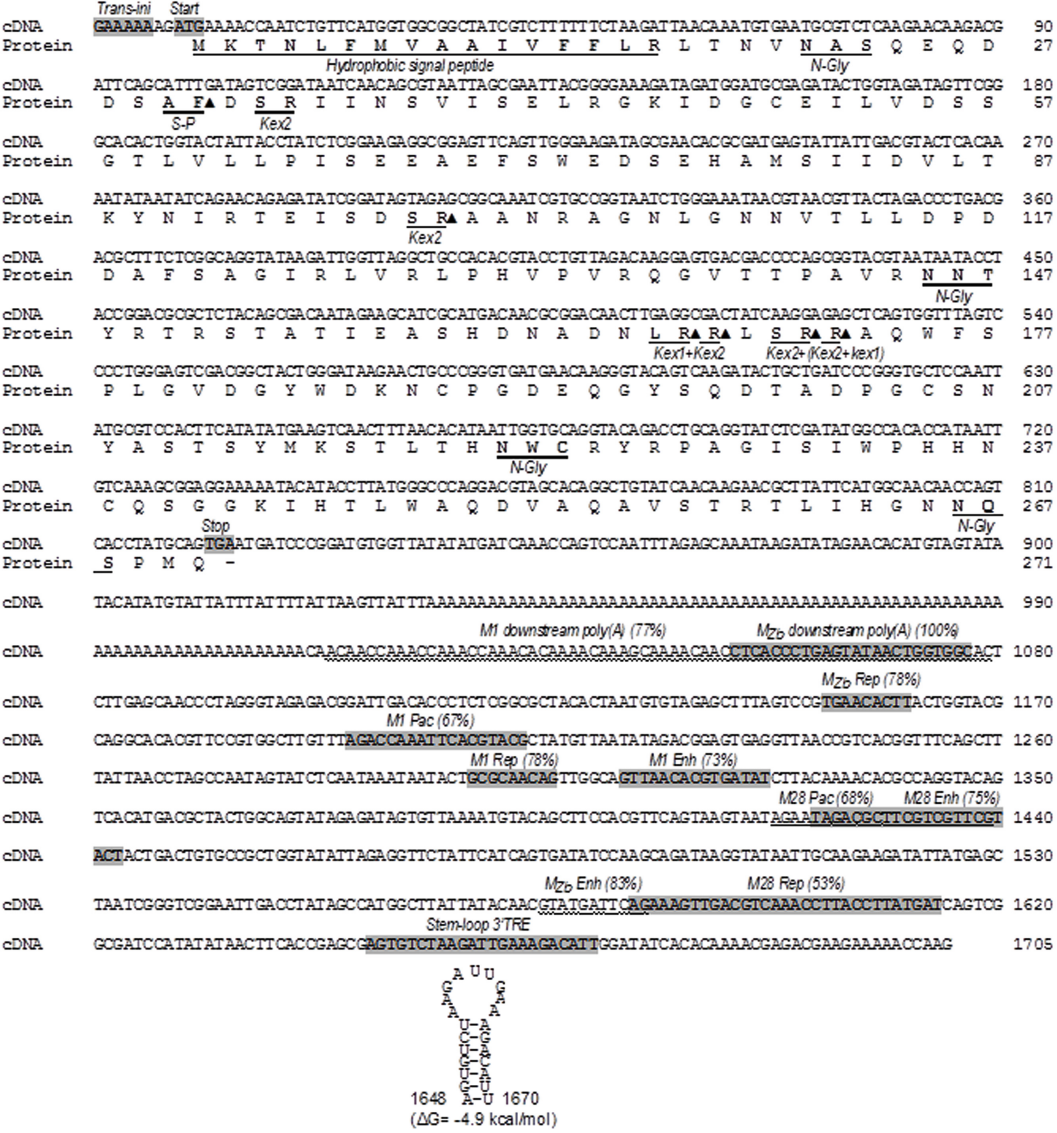
**Nucleotide sequence of the TdV-Mbarr-1 genome (cDNA) and the amino acid sequence of the ORF of the putative Kbarr-1 preprotoxin.** The Kbarr-1 amino acid sequence is displayed under the nucleotide sequence. The 5′ GAAAAA motif probably required for transcription initiation (trans-ini), the protein synthesis initiation codon (start), the protein synthesis stop codon (stop), the putative 3′-terminal recognition element for virus replication (stem-loop 3′ TRE) with a free energy of ΔG = –4.9 kcal/mol, the two regions homologous to ScV-M1 and M*_Zb_* dsRNAs located downstream from the central poly(A) [M1 downstream poly(A) and M*_Zb_* downstream poly(A)], and the eight regions homologous to previously reported replication signals of ZbV-M*_Zb_* (M*_Zb_* Rep), ScV-M1 (M1 Rep), and ScV-M28 (M28 Rep), internal replication enhancers of ScV-M1 (M1 Enh), ScV-M28 (M28 Enh), and ZbV-M*_Zb_* (M*_Zb_* Enh), or packaging signals of ScV-M1 (M1 Pac) and ScV-M28 (M28 Pac) are shown shaded in the nucleotide sequence (except for ScV-M1 downstream poly(A), ScV-M28 (M28 Pac) and ZbV-M*_Zb_* (M*_Zb_*-Enh) which are wavy underlined). The percentage of sequence identity is given in parentheses. The secondary structure of the putative *cis* signal for replication of TdV-Mbarr-1 RNA is at the bottom of the sequence. The hydrophobic signal peptide, the putative signal peptidase processing site (S-P), the putative Kex2 endopeptidase (Kex2) or Kex1 exopeptidase (Kex1) sites, and the potential N-glycosylation sites (N-Gly) are underlined in the amino acid sequence. Closed triangle, cleavage site. Sc, *Saccharomyces cerevisiae*; Zb, *Zygosaccharomyces bailii*.

The TdV-Mbarr-1 dsRNA band present in the Kbarr-1 strain EX1180 was purified after agarose-gel electrophoresis and sequenced by NGS techniques (see Materials and Methods). The full cDNA sequence determined was 1705 nucleotides, which is the same size as that estimated by agarose-gel electrophoresis, 1.7 kb (**Figures 3** and **4**; the 5′–3′ orientation refers to the positive strand with coding capacity). There is a 5′ GAAAAA conserved motif previously found in the ScV L and M genomes and probably required for transcription initiation (Fujimura and Wickner, 1989), followed by an open reading frame for the putative preprotoxin of 271 amino acids in the 5′-most region, from nucleotides 9 to 821. The central part of the RNA molecule contains an A-rich region with 78 adenine residues. The non-coding 3′-region is presumed to provide structural *cis*-elements required for RNA replication and encapsidation. A putative 3′-terminal recognition element (3′-TRE) from nucleotides 36 to 58 (numbering from the 3′ end) with a free energy of ΔG = -4.9 kcal/mol was found; although it has no significant identity with previously described 3′-TRE elements required for ssRNA replication of *S. cerevisiae* viral RNAs. No putative viral binding site (VBS) with typical stem loop structure interrupted by an unpaired protruding A residue was found in this 3′-region, despite these typical VBSs having been reported in the same genome region of LA, M1, and M28 dsRNAs, but not in Mlus (Rodríguez-Cousiño et al., 2011), and being required for ssRNA binding to Gag-Pol protein and packaging (Wickner et al., 2013). However, 10 sequence stretches homologous to previously reported poly(A) downstream regions (including regions of replication, replication-enhancement, and packaging signals) of several *S. cerevisiae* and *Z. bailii* M viral genomes were found in this TdV-Mbarr1 non-coding 3′-region, but not in the rest of the molecule (**Figure 4**). Apart from these similarities, the TdV-Mbarr-1 genome presented no relevant sequence identity with the known M virus genomes of yeasts, which, in terms of their overall sequences, themselves share no relevant sequence identity.

The ORF located in the 5′ region of Mbarr-1 contains a stretch of hydrophobic amino acids at the amino terminus according to the Kyte-Doolittle hydrophilicity plot (Kyte and Doolittle, 1982). This is a possible N-terminal secretion signal with a potential signal for peptidase cleavage. The ORF also presents six Kex2p/Kexlp processing sites and four potential sites for N-glycosylation (**Figure 4**). However, as it was the case for the genomes, the Kbarr-1 amino acid sequence showed no relevant sequence identity with the known yeast killer protein toxins, which, in terms of their overall sequences, themselves share no relevant amino acid sequence homology.

## Discussion

### Characterization of the New Kbarr-1 Killer Yeasts

The wine *T. delbrueckii* killer yeasts are less frequently isolated from spontaneous must fermentations than other wine killer yeasts such as *S. cerevisiae* K2 or Klus (Maqueda et al., 2012). This is probably because *T. delbrueckii* has slower growth rate and less fermentation vigor than *S. cerevisiae* yeasts in the grape must medium, so that *S. cerevisiae* quickly overcomes *T. delbrueckii* at the beginning of must fermentation. Also, isolation of the less frequent *T. delbrueckii* yeast may be missed because it resembles very much the colony morphology of *S. cerevisiae*, with the two being easily confused. We have succeeded probably because the intense killer phenotype of the new *T. delbrueckii* Kbarr-1 yeasts allowed them to dominate the tumultuous and end stages of the spontaneous must fermentation that we analyzed, facilitating their isolation. As they can kill other *S. cerevisiae* strains as well as many other yeast species, they may have a beneficial effect during food fermentation, as it has been shown for K2 killer strains in winemaking (Pérez et al., 2001). This is especially relevant for the use of *Torulaspora* in winemaking because this species is strongly recommended by winemaking supply companies to improve wine quality, while the non-killer strains are quickly displaced by the always present *S. cerevisiae* yeasts during must fermentation. These Kbarr-1 yeasts may also be useful to avoid undesirable potential spoilage and pathogen yeasts in food fermentation processes because they have the broadest antifungal spectrum hitherto described (**Figures 1** and **2**), even broader than that of the recently described Klus killer yeasts (Rodríguez-Cousiño et al., 2013), or the other *S. cerevisiae* killer yeasts (K1, K2, or K28) that mostly kill sensitive cells of the same or some congeneric species (Magliani et al., 1997). Although the intensity of the Kbarr-1 killer activity may change depending on the assay conditions (pH, temperature, and the sensitive strain tested) as it happen for other killer toxins (Pfeiffer and Radler, 1984; Young, 1987), it is active against different yeast species in the typical food fermentation environment: acidic (pH 3.5–5.5) and mild temperature (18–28°C).

### Genetic Characterization of the Mbarr-1 Virus

The Kbarr-1 killer yeasts contained two dsRNA molecules corresponding to a new TdV-Mbarr-1 dsRNA and the genome of its putative helper virus TdV-LAbarr, as found in other killer yeasts (Fujimura and Esteban, 2011, 2013; Rodríguez-Cousiño et al., 2011, 2013). The putative dependency of TdV-Mbarr-1 on TdV-LAbarr (and/or TdV-LBC) is likely because we never found Mbarr-1 killer yeasts without the 4.6 kb dsRNA among the 152 isolated yeasts. We never found killer Kbarr-1 yeast free of the 1.7 kb dsRNA, suggesting that the Kbarr-1 toxin is encoded by this RNA molecule. Moreover, this was confirmed by the Kbarr-1 yeasts becoming non-killer and sensitive to Kbarr-1 killer yeasts when they lost the 1.7 kb dsRNA after cycloheximide treatment.

### Mbarr-1 dsRNA Organization and the Encoded Kbarr-1 Preprotoxin

**FIGURE 5:**
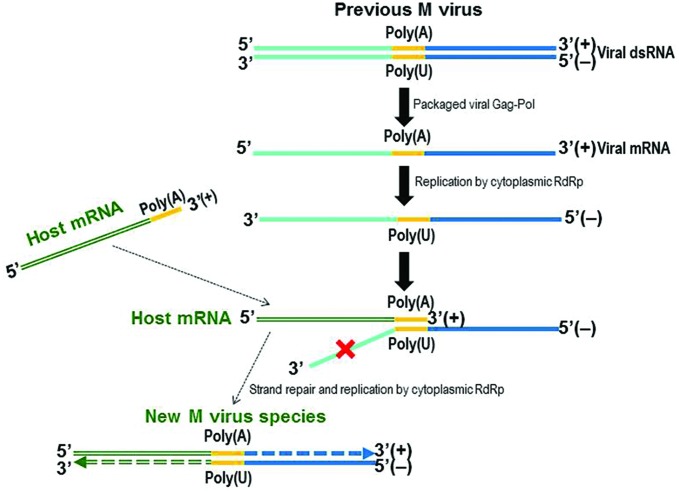
**Model of the emergence of the M virus species.** Free mRNA (+) might be replicated to generate a ssRNA(-) instead of a natural dsRNA molecule, by a cytoplasmic RdRp that could be Pol of Gag-Pol of the LA virus. Complementary base pairing of the ssRNA(-) central poly(U) and the 3′ poly(A) of a host mRNA(+) may generate a new incomplete hybrid dsRNA molecule that could be repaired and replicated by the RdRp to yield a new dsRNA M virus.

The complete cDNA sequence of Mbarr-1 dsRNA (1705 nucleotides) matches the Mbarr-1 dsRNA size estimated by agarose-gel electrophoresis (1.7 kb, **Figure 4**), and it includes the 5′ and 3′ ends, and the central A-rich region previously found in the *S. cerevisiae* M viruses. This suggests that the NGS techniques used here for dsRNA sequencing yielded more satisfactory results than those we used previously for ScV-Mlus dsRNA sequencing, where the length of the sequence obtained (2033 nucleotides) was shorter than the 2.3 kb visualized on agarose gel electrophoresis (Rodríguez-Cousiño et al., 2011). This difference was explained as due to the variable number of adenine residues in the central A-rich region, which supposedly accounts for the different-sized ScV-Mlus isotypes. This central A-rich region may facilitate sliding or jumping of either the reverse transcriptase or the Taq polymerase used in RT-PCR, yielding a slightly shorter sequence than the actual. This methodological problem is overcome by using the new NGS sequencing approach, given that it yields the same sequence length as the actual dsRNA size. TdV-Mbarr-1 contains a single continuous central A-rich region as do most of M dsRNAs (Wickner, 1996) with the exception of ScV-Mlus which contains two central A-rich regions of variable size (Rodríguez-Cousiño et al., 2011). Additionally, Mbarr-1 also resembles the ScV-M genomes in that it contains the conserved 5′ GAAAAA motif probably required for transcription initiation, a putative 3′-TRE with low free energy, and 10 sequence stretches homologous to previously reported poly(A)-downstream-regions considered as putative signals for replication, replication-enhancement, and packaging, located in the non-coding 3′-region of the *S. cerevisiae* and *Z. bailii* M viral genomes (Weiler et al., 2002; Wickner et al., 2013; **Figure 4**). Overall, the genome organization of Mbarr-1 dsRNA resembles that of other toxin-encoding satellite viruses such as ScV-M1, ScV-M2, ScV-M28, ScV-Mlus, or *Z. bailii* M*_Zb_* dsRNA (Schmitt and Breinig, 2002; Weiler et al., 2002; Rodríguez-Cousiño et al., 2011). The conserved motifs located in the non-coding 3′-region suggest a close phylogenic relationship of *T. delbrueckii*, *S. cerevisiae*, and *Z. bailii* M viruses. The coding 5′-region of the M viruses may have an independent origin, probably from host yeast genes coding for secreted proteins, given that a relevant amino acid sequence identity between the *S. cerevisiae* Klus toxin and the nuclear YFR020W ORF protein has been found (Rodríguez-Cousiño et al., 2011). Probably, the central A-rich region located between the coding 5′-region and the non-coding 3′-region of the M viruses is just a reminiscence of the mRNA 3′ poly(A)-tail of a hypothetically secreted and chromosomally encoded protein, which somehow became attached to the 5′ end of the viral-RNA, which in turn formed the non-coding 3′-region of the final M RNA. Thereafter, as shown in **Figure 5**, new M viruses species can be generated by annealing of the 3′ poly(A)-tail of other incoming mRNA with the central poly(U) of a pre-existing viral M ssRNA(-), followed by a conservative replication of the complementary strands by a cytoplasmic RdRp to create a distinct “5′ host mRNA-central poly(A)-3′ viral RNA” hybrid RNA molecule. The RdRp required for this conservative replication could be provided by Pol of the Gag-Pol fusion protein of LA helper virus. Further studies will be required to confirm this hypothesis.

The AUG initiation codon of the putative Mbarr-1 preprotoxin ORF is located close to the 5′ end of the RNA molecule (**Figure 4**), like in M1, M28, and M*_Zb_* RNAs (Skipper et al., 1984; Meskaukas, 1990; Schmitt and Tipper, 1995; Weiler et al., 2002) but different to the Mlus RNA whose AUG codon is located 112 nt away from the 5′ end (Rodríguez-Cousiño et al., 2011). Also as for other M dsRNA, the Mbarr-1 ORF organization resembles that of other killer preprotoxins such as those of M1, M2, M28, or Mlus viruses. It contains a stretch of hydrophobic amino acids at the amino terminus, potential Kex2p/Kexlp processing sites, and potential sites for N-glycosylation (**Figure 4**). Proteolytic cleavage of the Kbarr-1 preprotoxin by signal peptidase and Kex2/Kex1 proteases could produce four putative peptides, the signal peptide and the α, β, and γ subunits. According to the disulphide bond prediction (Ceroni et al., 2006), there is a potential disulphide bond between cysteine-50, located in the putative α subunit, and cysteine-189 or cysteine-238, both located in the putative β subunit of the Kbarr-1 mature toxin. This disulphide bond may be required for keeping α and β subunits together during the preprotoxin processing and secretion to yield an extracellular active α/β heterodimer.

## Conclusion

The new *T. delbrueckii* Kbarr-1 wine yeasts kill all the tested *S. cerevisiae* strains as well as many other undesired yeast species in environmental conditions similar to those of the typical sugar-rich substrate fermentation, which makes them an interesting starter culture for the food fermentation industry. The killer dsRNA virus system of this wine yeast seems very similar to those previously described in *S. cerevisiae*. Although there was no relevant general sequence identity among the M genomes, the relevant identity found in 10 sequence stretches of TdV-Mbarr-1 RNA with previously reported M RNAs raises the possibility that the M viruses may have a common phylogenic origin, at least for the non-coding 3′-region where these homologous sequences are located.

## Author Contributions

MR conceived the project, MR, RV, and MM designed and performed the experiments, MR, AL-P, and JR analyzed the data, MR wrote and edited the manuscript.

## Conflict of Interest Statement

The authors declare that the research was conducted in the absence of any commercial or financial relationships that could be construed as a potential conflict of interest.

## Footnotes

1 http://www.technelysium.com.au/chromas.html

2 http://ncbi.nlm.nih.gov
